# The relationship between blood-based tumor mutation burden level and efficacy of PD-1/PD-L1 inhibitors in advanced non-small cell lung cancer: a systematic review and meta-analysis

**DOI:** 10.1186/s12885-021-08924-z

**Published:** 2021-11-13

**Authors:** He Ba, Lei Liu, Qiang Peng, Jie Chen, Yao-dong Zhu

**Affiliations:** 1 Department of Integrated Traditional and Western Medicine in Oncology, First Affiliated Hospital of Medical University of Anhui, Anhui, China; 2 Department of Hepatobiliary Surgery, First Affiliated Hospital of Medical University of Anhui, Anhui, China; 3 Department of Gastrointestinal Surgery, First Affiliated Hospital of Medical University of Anhui, Anhui, China

**Keywords:** PD-1, PD-L1, NSCLC, Tumor mutation burden, Blood, Lung cancer, Meta-analysis, Survival

## Abstract

**Background:**

The predictive role of blood-based tumor mutation burden (bTMB) for selecting advanced nonsmall cell lung cancer (NSCLC) patients who might benefit from immune checkpoint inhibitors (ICIs) is still under debate. Therefore, the purpose of this meta-analysis was to evaluate the efficacy of programmed cell death 1 (PD-1) /programmed cell death ligand 1 (PD-L1) inhibitors versus that of standard-of-care therapy in patients with NSCLC who were bTMB high and bTMB low.

**Methods:**

PubMed, Embase, Cochrane, the Web of Science, and ClinicalTrials.gov were searched systematically from inception to February 2021 for studies of PD-1/PD-L1 inhibitors (durvalumab OR atezolizumab OR avelumab OR pembrolizumab OR Nivolumab) that provided hazard ratios (HRs) for overall survival (OS) or progression-free survival (PFS), or odds ratios (ORs) for objective response rate (ORR) in both bTMB high and bTMB low groups.

**Results:**

A total of 2338 patients with advanced or metastatic NSCLC from six randomized controlled trials, which all used chemotherapy (CT) as a control, were included in this study. Compared with CT, PD-1/PD-L1 inhibitor therapy improved OS (HR 0.62, 95% CI 0.52–0.75, *P* < 0.01), PFS (HR 0.57, 95% CI 0.48–0.67, *P* < 0.01), and ORR (OR 2.69, 95% CI 1.84–3.93, *P* < 0.01) in bTMB-high NSCLC patients but not in bTMB-low patients (OS HR 0.86, 95% CI 0.69–1.07, *P* = 0.17; PFS HR 1.00, 95% CI 0.78–1.27, *P* = 0.98; ORR OR 0.63, 95% CI 0.49–0.80, *P* = 0.03). Subgroup analyses showed that these results were consistent across all subgroups (line of therapy, therapy regimen, type of NGS panel, PD-L1 expression, and cutoff value). Meta-regression analysis showed that the proportion of patients with squamous cell histology had no statistical effect on clinical outcomes. Sensitivity analyses illustrated that all results were stable.

**Conclusions:**

The efficacy of PD-1/PD-L1 inhibitor therapy in advanced NSCLC patients may be dependent on bTMB level. Patients with high bTMB tend to obtain significantly better OS, PFS, and ORR from PD-1/PD-L1 inhibitor therapy than from CT. However, because of multiple limitations, including those related to reproducibility, the results are exploratory and should be interpreted with caution.

**Supplementary Information:**

The online version contains supplementary material available at 10.1186/s12885-021-08924-z.

## Background

In the past few years, programmed cell death 1 (PD-1)/programmed cell death ligand 1(PD-L1) inhibitor therapy has replaced chemotherapy (CT) as the new standard second- or later-line therapy for many tumors. Different from conventional CT, PD-1/PD-L1 inhibitors can increase the activation of immune cells by blocking the PD-1/PD-L1 pathway and lead to immune-mediated tumor cell clearance, resulting in superior efficacy and fewer adverse effects than conventional CT [1]. However, only a fraction of patients can benefit from PD-1/PD-L1 inhibitor therapy, and some of them may suffer from immune-related side effects [2, 3]. In addition, the cost of PD-1/PD-L1 therapy is approximately 300,000 dollars per year, which is high [4]. Thus, the identification of biomarkers that are adequate to screen patients more likely to experience greater efficacy is necessary. At present, the expression of PD-L1 is recognized as the most plausible and available biomarker for the selection of patients who may benefit from the PD-1/PD-L1 inhibitor therapy [5]. However, several trials have reported favorable outcomes in PD-L1-negative patients, indicating that PD-L1 alone is not adequate for the achievement of comprehensive and accurate screening [6, 7]. Therefore, more biomarkers that can be used to screen the population that would benefit from PD-1/PD-L1 inhibitor therapy are urgently needed.

Tumor mutation burden (TMB), defined as the total number of somatic mutations per megabase of interrogated genomic sequence, is recognized as a biomarker independent of PD-L1 [8]. It was reported that tumors with a higher TMB can carry more mutated genes that may produce new antigens, which are able to enhance the probability of immune cell recognition of tumor cells; thus, patients with a high TMB tend to obtain a better curative effect from PD-1/PD-L1 inhibitors [9, 10]. Clinical trials also suggested that patients with high tissue-based TMB (tTMB) can obtain better efficacy from PD-1/PD-L1 inhibitor therapy for nonsmall cell lung cancer (NSCLC), melanoma and gastric cancer [11–13].

Lung cancer has the highest morbidity and mortality rates worldwide. NSCLC, the most common subtype of lung cancer, accounting for over 80% cases [14]. The exploration of biomarkers for use in NSCLC immunotherapy has drawn wide attention among clinicians and researchers. It has been reported that cancers related to environmental chronic DNA damage tend to exhibit higher TMB [15]. Lung cancer has almost the highest tTMB among solid tumors and is believed to be associated with direct exposure to mutagens in tobacco smoking [16]. Several meta-analyses have reported that tTMB may serve as a predictive biomarker for immune checkpoint inhibitors (ICIs) in NSCLC, and even the predictive value of tTMB for long-term survival in NSCLC patients is still disputed [17–19]. However, there are several limitations in tTMB detection. First, the clinical detection of tTMB is limited by the challenge of obtaining enough tissue with adequate quality from patients. According to statistics, approximately 30% of NSCLC patients cannot provide enough tissue or are not suitable for tTMB detection [20]. Second, the invasive nature of tissue biopsy also makes tTMB unable to be used in the dynamic monitoring of cancer therapy. In addition, the tTMB level in tumors is not uniform, and the result of tTMB detection may be affected by sampling location and tumor histology [21].

In recent years, with the development of liquid biopsy and next-generation sequencing (NGS) technology, detecting TMB noninvasively based on circulating tumor deoxyribonucleic acid (ctDNA) in blood has become a reality and, greatly increases the feasibility, sensitivity and dynamics of TMB detection. Moreover, compared with single point biopsy, blood detection is not susceptible to potential sampling bias, which can reduce the impact of tumor tissue heterogeneity on TMB testing results. Several studies have reported that NSCLC patients with high blood-based TMB (bTMB) could obtain better efficacy from PD-1/PD-L1 inhibitor therapy than patients whose bTMB is low [22–25]. However, opposite results were also reported by a nonnegligible number of studies [26, 27]. Moreover, although studies have consistently reported better efficacy of PD-1/PD-L1 inhibitors than of CT in NSCLC patients who were bTMB high, it is still unclear which kind of therapy can bTMB-low patients more benefits. To address these concerns, we performed a meta-analysis to explore the relationship between bTMB levels and the relative efficacy of PD-1/PD-L1 inhibitors in NSCLC patients with high and low bTMB.

## Methods

This meta-analysis was performed in accordance with the Preferred Reporting Items for Systematic Reviews and Meta-analyses (PRISMA), and has been registered in PROSPERO. The registration code is CRD42021233992.

### Search strategy

Two investigators independently searched PubMed, Embase, Cochrane, the Web of Science and ClinicalTrials.gov from inception to February 2021 for phase II and phase III randomized controlled trials (RCTs). The main search terms were as follows: (immunotherapy OR immunization OR Immune Checkpoint Inhibitor OR immune checkpoint blocker OR ICI OR ICB OR PD-1/PD-L1 OR durvalumab OR atezolizumab OR avelumab OR pembrolizumab OR Nivolumab) AND (mutation burden OR mutational burden OR mutation load OR mutational load OR TMB OR TML) AND (cell free OR circulating OR extracellular OR blood OR plasma OR serum OR liquid biopsy OR cirDNA OR ctDNA OR cfDNA) AND (NSCLC OR non small cell OR lung cancer). The references of relevant studies and reviews were also manually searched for more eligible trials. The search strategy is provided in the supplementary materials.

### Study selection

RCTs that met the following criteria were enrolled: (P) patients with advanced or metastatic NSCLC whose bTMB levels were evaluable and who were divided into bTMB-high and bTMB-low groups according to the cutoff value. (I): Patients in the experimental arm were treated with PD-1/PD-L1 inhibitors alone or in combination with other anticancer agents, including CTLA-4inhibitors, CT and targeted agents. (C): Patients in the control arm were treated with standard of care therapy. (O): The hazard ratios (HRs) of overall survival (OS) or progression-free survival (PFS) or odds ratios (ORs) of the objective response rate (ORR) and their 95% confidence intervals (95%CIs) were reported according to bTMB level in the study. When duplicate publications were identified, only the most recent and comprehensive publication was included.

All reviews, editorials, letters, comments, trial designs, conference abstracts, basic studies and irrelevant clinical trials were excluded. Studies that did not provide therapy outcomes stratified by bTMB, and studies that reported only subgroup efficacy analyses among bTMB-high or bTMB-low patients only were also excluded.

### Data extraction

The following information was extracted from every trial by two investigators independently: first author, year of publication, trial design, number of bTMB-high and bTMB-low patients in the experimental and control arms, therapy regimen in both arms, line of therapy, PD-L1 selection status, sex and median age of patients in both arms, residence area of patients, type of nest-generation sequencing (NGS) panel, bTMB cutoff value, median follow-up time, and value and 95% CI of outcomes (ORR, PFS, OS) in the bTMB-high and bTMB-low group. Inconsistencies were conferred and resolved by consensus among all investigators.

### Quality assessment

The Cochrane Risk of Bias Tool was applied for the assessment of risk of bias. The following six criteria in every RCT were examined and categorized as high risk, low risk, or unclear risk: sequence generation, allocation concealment, blinding, incomplete outcome data, selective reporting, and other bias. Data extraction and quality assessment were carried out by two investigators independently, and any disagreements were resolved by discussion and consensus among all investigators.

### Statistical analysis

The primary endpoint was the difference in the efficacy of PD-1/PD-L1 inhibitors measured by HR of OS and PFS, and OR of ORR between the experimental and control arms in bTMB-high and bTMB-low groups. If the heterogeneity test showed significant heterogeneity (*I*^2^ > 50%, *P* < 0.1), a random-effects model was used to calculate the pooled OR and HR in bTMB-high and bTMB-low patients; otherwise, a fixed-effects model was applied. *P* < 0.05 was defined as a statistically significant outcome. Heterogeneity among individual studies was assessed by the Q-test, and the result was qualified by the *I*^*2*^ statistic and *P* value. *I*^*2*^ > 50% and/or *P* < 0.1 were considered to indicate significant heterogeneity. To explore the source of heterogeneity, subgroup analyses stratified by line of therapy, therapy regimen, type of NGS panel, PD-L1 selection status, and bTMB cutoff value were performed. To assess the effect of histology on the pooled clinical outcomes, a meta-regression was conducted using the “metareg” command, and the threshold for statistical significance was set at *P* < 0.05. Sensitivity analyses were also performed to test the stability of the results in this study. Publication bias was not assessed because only 6trials were enrolled in this study, and the funnel plot cannot detect publication bias effectively when the number of studies included is less than ten [28]. All analyses were carried out by Stata version 15.0 (Stata Corporation, College Station, TX), and HR and OR were calculated by SPSS 22.0 (IBM Corporation).

## Results

### Eligible studies and characteristics

**Fig. 1:**
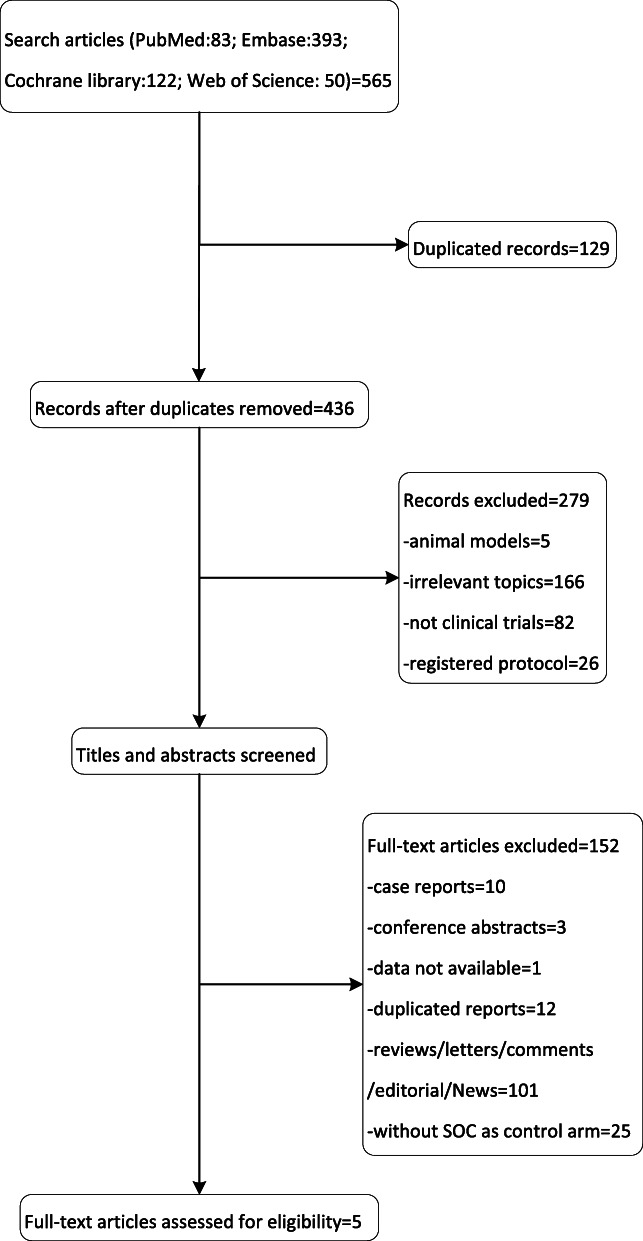
Flow chart of systematic literature search

A total of 565 records were included in the initial assessment by searching the literature database and references of relevant studies. After assessment, fsix studies, including 2338 patients with advanced or metastatic NSCLC, were involved in this systematic review and meta-analysis (Fig. 1) [29–33].

All trials, except POPLAR, which is a phase II trial, were multicenter phase III trials published within 3 years. In all studies, the level of bTMB was defined as the number of mutations per megabase of ctDNA. Among the patients included, 741 patients (31.7%) were bTMB high, and 1597 patients (68.3%) were bTMB low. The POPLAR, OAK, MYSTIC, and KEYNOTE-189 studies reported data on tTMB and the correlation of bTMB and tTMB in some of the enrolled patients. Cohorts in the POPLAR and OAK studies were mixed oncogene-addicted and nonaddicted patients, while only oncogene-nonaddicted patients were included in the remaining studies. The BGB-A317–307 study enrolled patients with squamous cell cancer only, KEYNOTE-189 included patients with nonsquamous cell cancer only, and cohorts in the remaining studies were composed of squamous and nonsquamous cell cancer patients. Different PD-1/PD-L1 inhibitors were used in the intervention arm, including 4 trials that used PD-L1 inhibitors as monotherapy (atezolizumab, durvalumab), 2 trials that used PD-1 inhibitors plus CT (pembrolizumab plus CT, tislelizumab plus CT), and 1 trial that used a PD-L1 inhibitor plus a CTLA-4 inhibitor (durvalumab plus tremelimumab). All trials used CT in the control arm. Except for the Impower110 trial, all trials chose patients unselected for PD-L1 as research subjects. In terms of line of therapy, four trials were performed in first-line settings, and two were performed in subsequent settings. The characteristics of the six eligible trials are presented in Table 1.

**Table 1:** Characteristics and outcomes of enrolled trials

Study ID	Year	First author	Trial design	No. patients						Cut-off (mut/meg)
				bTMB high			bTMB low			
				Exp		Con	Exp		Con	
**POPLAR**	2018	Gandara DR	Phase II open-label	25		38	80		68	16
**OAK**	2018	Gandara DR	Phase III open-label	158 (BEP)			425 (BEP)			16
**MYSTIC**	2020	Rizvi, N. A.	Phase III open-label	77		70	209		185	20
				64		70	204		185	20
**IMpower110**	2020	Herbst, R. S.	Phase III open-label	87 (BEP)			302 (BEP)			16
**BGB-A317–307**	2020	Wang, J.	Phase III open-label	48		14	33		16	6
**KEYNOTE-189**	2020	Garassino, M. C.	Phase III double blinded	70		28	90		47	15
**Study ID**	**No. patients (tTMB available)**					**tTMB Cut-off (mut/meg)**				
	**tTMB high**		**tTMB low**							
	**Exp**	**Con**	**Exp**	**Con**						
**POPLAR&OAK**	88 (BEP)		171 (BEP)			16				
**MYSTIC**	60	85	67	84		10				
	60	104	67	84		10				
**IMpower110**	NR	NR	NR	NR		NA				
**BGB-A317–307**	NR	NR	NR	NR		NA				
**KEYNOTE-189**	100	34	107	52		10				
**Study ID**	**No. of patients with matched samples for bTMB and tTMB**	**correlation between tTMB and bTMB**	**Mutation in the cohort**	**Mutation target**						
**POPLAR&OAK**	259	Spearman ρ = 0.64	mixed	EGFR, KRAS, EML4-ALK						
**MYSTIC**	352	Pearson r = 0.70	none	NA						
**IMpower110**	NA	NA	none	NA						
**BGB-A317–307**	NA	NA	none	NA						
**KEYNOTE-189**	235	Pearson r = 0.61	none	NA						
**Study ID**	**Clinical stage**	**Histology (squamous)**								
		**Total**	**Exp**	**Con**			**bTMB low**	**bTMB high**		
**POPLAR**	IIIB-IV	76 (36%)	38 (36%)	38 (36%)			56 (38%)	20 (32%)		
**OAK**	IIIB-IV	176 (30%)	87 (30%)	89 (31%)			121 (28%)	55 (35%)		
**MYSTIC**	IV	164 (30%)	88 (31%)	76 (30%)			106 (27%)	58 (39%)		
	IV	158 (30%)	82 (31%)	76 (30%)			104 (27%)	54 (40%)		
**IMpower110**	IV	117 (30%)	NR	NR			NR	NR		
**BGB-A317–307**	IIIB-IV	111 (100%)	81 (100%)	30 (100%)			49 (100%)	62 (100%)		
**KEYNOTE-189**	IV	0 (0%)	0 (0%)	0 (0%)			0 (0%)	0 (0%)		
**Study ID**	**Area**	**Male, No. (%)**		**Median age**				**Line of therapy**	**PD-L1 selection**	**NGS panel**
		**Exp**	**Con**	**Exp**			**Con**			
**POPLAR**	multiple areas	72 (69%)	58 (54.7%)	61			63	≥2	Unselected	FoundationOne
**OAK**	multiple areas	193 (65.9%)	183 (63.1%)	63			64	≥2	Unselected	FoundationOne
**MYSTIC**	multiple areas	202 (70.6%)	175 (68.6%)	67 (bTMB high)	64 (bTMB low)	63 (bTMB high)	64 (bTMB low)	1	Unselected	Guardant OMNI
	multiple areas	189 (71%)	175 (68.6%)	66 (high)	65 (low)	63 (high)	64 (low)	1	Unselected	Guardant OMNI
**IMpower110**	multiple areas	285 (BEP)	104 (BEP)	65 (BEP)		1	PD-L1 positive	FoundationOne		
**BGB-A317–307**	multiple areas	NR	NR	NR	NR	1	Unselected	OncoScreen Plus		
**KEYNOTE-189**	Asian	NR	NR	NR	NR	1	Unselected	Guardant OMNI		
**Study ID**	**Therapy regimen**						**Median follow-up (month)**			
	**Exp**		**Con**				**Exp**	**Con**		
**POPLAR**	Atezolizumab (1200 mg every 3 weeks)		Docetaxel (75 mg/m^2^ every 3 weeks)				14.8	15.7		
**OAK**	Atezolizumab (1200 mg every 3 weeks)		Docetaxel (75 mg/m^2^ every 3 weeks)				21	21		
**MYSTIC**	Durvalumab (20 mg/kg every 4 weeks)		investigator’s choice of platinum-based doublet chemotherapy				30.2	30.2		
	Durvalumab+ tremelimumab (20 mg/kg of durvalumab every 4 weeks+ 1 mg/kg of tremelimumab every 4 weeks)		investigator’s choice of platinum-based doublet chemotherapy				30.2	30.2		
**IMpower110**	Atezolizumab (1200 mg every 3 weeks)		investigator’s choice of platinum-based doublet chemotherapy				13.4–15.7	13.4–15.7		
**BGB-A317–307**	Tislelizumab+ Paclitaxel/(nab)- Paclitaxel+ carboplatin (Tislelizumab 200 mg + carboplatin AUC 5+ paclitaxel 175 mg/m^2^ / (nab)-paclitaxel 100 mg/m^2^ every 3 weeks)		paclitaxel 175 mg/m^2^ + carboplatin AUC 5 every 3 weeks				8.6	8.6		
**KEYNOTE-189**	Pembrolizumab+pemetrexed+cisplatin/ carboplatin (pembrolizumab 200 mg + pemetrexed 500 mg/m^2^ + cisplatin 75 mg/m^2^ OR carboplatin AUC 5 every 3 weeks)		Placebo+ pemetrexed+ cisplatin/ carboplatin (saline placebo+pemetrexed 500 mg/m^2^ + cisplatin 75 mg/m^2^ OR carboplatin AUC 5 every 3 weeks)				21	21		
**Study ID**	**HR for OS (95% CI), Exp vs Con**		**OR for ORR (95% CI), Exp vs Con**				**HR for PFS (95% CI), Exp vs Con**			
	**High**	**Low**	**High**		**Low**		**High**	**Low**		
**POPLAR**	0.56 (0.31–0.99)	0.76 (0.52–1.12)	4.38 (0.99–19.27)		0.48 (0.19–1.21)		0.57 (0.33–0.99)	1.09 (0.77–1.55)		
**OAK**	0.64 (0.44–0.92)	0.65 (0.52–0.81)	2.07 (0.82–5.20)		0.84 (0.49–1.43)		0.65 (0.47–0.92)	0.98 (0.80–1.20)		
**MYSTIC**	0.72 (0.50–1.05)	0.93 (0.74–1.16)	1.56 (0.74–3.36)		0.57 (0.36–0.89)		0.77 (0.52–1.13)	1.19 (0.94–1.50)		
	0.49 (0.32–0.74)	1.16 (0.93–1.45)	3.44 (1.65–7.46)		0.44 (0.27–0.71)		0.53 (0.34–0.81)	1.55 (1.23–1.94)		
**IMpower110**	0.75 (0.41–1.35)	1.07 (0.77–1.47)	NR		NR		0.55 (0.33–0.92)	1.00 (0.78–1.29)		
**BGB-A317–307**	NR	NR	4.04 (1.13–14.41)		0.63 (0.19–2.18)		0.30 (0.13–0.67)	0.63 (0.25–1.61)		
**KEYNOTE-189**	0.61 (0.36–1.06)	0.64 (0.41–0.99)	3.61 (1.50–9.00)		1.18 (0.58–2.41)		0.35 (0.21–0.57)	0.50 (0.34–0.73)		

***Exp*** **experimental group,** ***BEP*** **bTMB-evaluable population,** ***Con*** **control group,** ***mut/meg*** **mutation/mergbase** ***NR*** **not reported,** ***95% CI*** **95% confidence interval,** ***HR*** **hazard ratio,** ***OS*** **overall survival,** ***OR*** **odds ratio,** ***ORR*** **objective response rate,** ***PFS*** **progression free survival**

### Relationship between bTMB level and overall survival

**Fig. 2:**
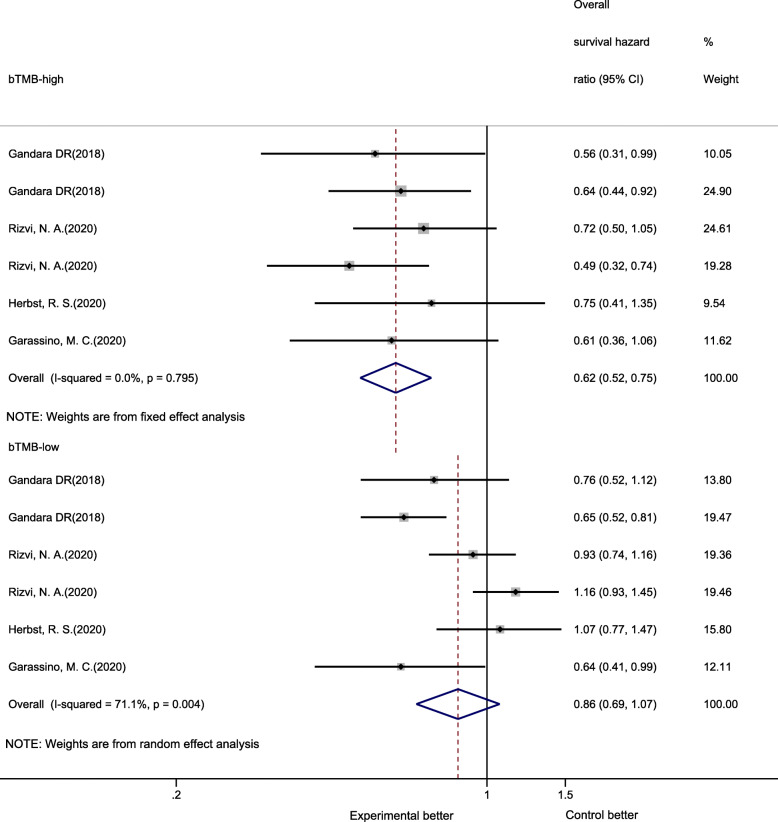
Forest plot of hazard ratios for overall survival comparing PD-1/PD-L1 inhibitors with chemotherapy

Five trials (six cohorts) that included 2227 NSCLC patients evaluated the correlation between bTMB and OS. Compared with CT, PD-1/PD-L1 inhibitor therapy reduced the risk of death in bTMB-high patients (HR 0.62, 95% CI 0.52–0.75, *P* < 0.01) but not in bTMB-low patients (OS HR 0.86, 95% CI 0.69–1.07, *P* = 0.17) (Fig. 2). Substantial heterogeneity was found among single studies for patients who were bTMB low (*I*^*2*^ = 71.1%, *P* < 0.01) but not in patients who were bTMB high (*I*^*2*^ = 0.00%, *P* = 0.80).

In the subgroup analysis of bTMB-high patients, across all subgroups (line of therapy, therapy regimen, type of NGS panel, PD-L1 expression, and cutoff value), patients receiving PD-1/PD-L1 inhibitors had significantly improved OS compared with patients receiving CT; moreover, the OS advantage achieved from PD-1/PD-L1 inhibitor therapy was larger in the bTMB-high group than in the bTMB-low group. The subgroup analysis of bTMB-low patients showed that regardless of therapy regimen, type of NGS panel, PD-L1 expression and cutoff value, PD-1/PD-L1 inhibitor therapy could reduce the risk of death in comparison with the outcomes associated with CT, but the difference was not statistically significant. However, a significant OS benefit was noted in second- or later-line of therapy (Table 2).

**Table 2:** Subgroup analyses of OS in bTMB high and bTMB low NSCLC patients

Variable	No. of cohorts	No. of patients	bTMB high			bTMB low		
		bTMB high /bTMB low	Pooled HR (95% CI)	***P***	***I***^**2**^ (%)	Pooled HR (95% CI)	***P***	***I***^**2**^ (%)
**Line of therapy**								
1	4	466/1222	0.63 (0.50–0.79)	< 0.01	0	0.97 (0.79–1.19)	0.79	51.8
≥2	2	221/573	0.62 (0.45–0.84)	< 0.01	0	0.68 (0.56–0.82)	< 0.01	0
**Therapy regimen**								
Anti-PD-L1 vs CT	4	455/1269	0.67 (0.54–0.83)	< 0.01	0	0.83 (0.66–1.05)	0.12	63.3
Anti-PD-L1 plus anti-CTLA-4 vs CT	1	134/389	0.49 (0.32–0.75)	< 0.01	N/A	1.16 (0.93–1.45)	0.19	N/A
Anti-PD-1 plus CT vs CT	1	98/137	0.61 (0.36–1.05)	0.07	N/A	0.64 (0.41–0.99)	0.05	N/A
**Type of NGS panel**								
Foundation one	3	308/875	0.64 (0.49–0.85)	< 0.01	0	0.80 (0.58–1.09)	0.15	67.8
Gardant OMNI	3	379/920	0.61 (0.48–0.78)	< 0.01	0	0.93 (0.71–1.23)	0.62	66.7
**PD-L1 expression**								
Unselected for PD-L1	5	600/1493	0.61 (0.50–0.74)	< 0.01	0	0.82 (0.65–1.05)	0.12	74.5
PD-L1 positive	1	87/302	0.75 (0.41–1.36)	0.34	N/A	1.07 (0.77–1.48)	0.68	N/A
**Cut-off value (mut/meg)**								
16	3	308/875	0.64 (0.49–0.85)	< 0.01	0	0.80 (0.58–1.09)	0.15	67.8
Others	3	379/920	0.61 (0.48–0.78)	< 0.01	0	0.93 (0.71–1.23)	0.62	66.7

***mut/meg*, mutation/mergbase,** ***N/A*** **not applicable,** ***95% CI*** **95% confidence interval,** ***HR*** **hazard ratio, OS overall survival**

### Relationship between bTMB level and progression-free survival

**Fig. 3:**
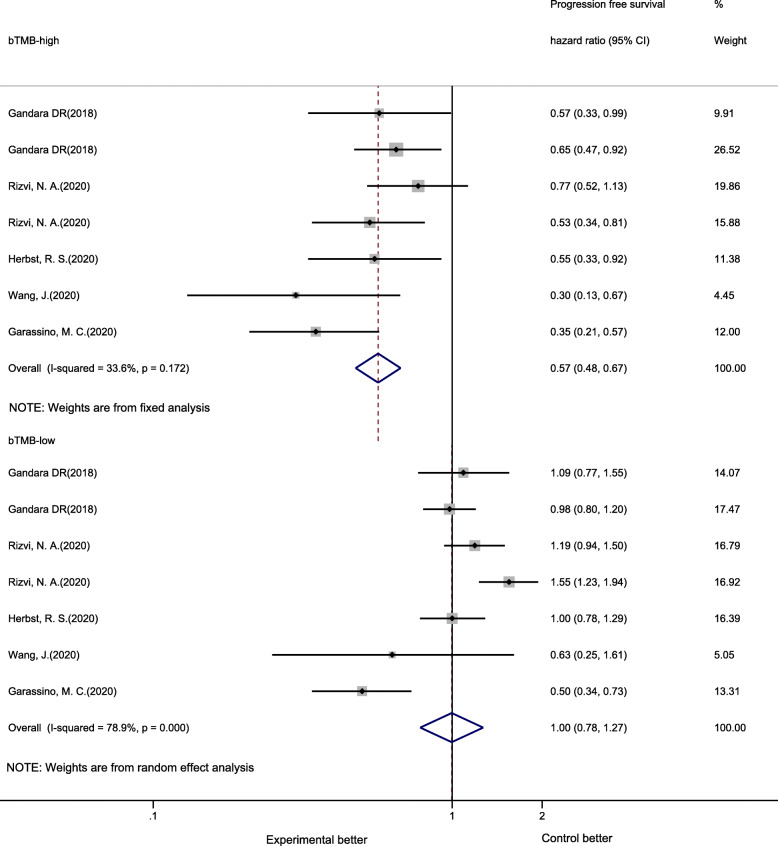
Forest plot of hazard ratios for progression-free survival comparing PD-1/PD-L1 inhibitors with chemotherapy

Six trials (seven cohorts) that included 2338 NSCLC patients assessed the relationship between bTMB and PFS of PD-1/PD-L1 inhibitor therapy. Patients with high bTMB showed a notably superior PFS with treated with PD-1/PD-L1 inhibitor therapy than with CT (PFS HR 0.57, 95% CI 0.48–0.67, *P* < 0.01), while in bTMB-low patients, no difference in PFS between PD-1/PD-L1 inhibitor therapy and CT was found (PFS HR 1.00, 95% CI 0.78–1.27, *P* = 0.98) (Fig. 3). Among single-study estimates in bTMB-high patients, no significant heterogeneity (Q = 9.03, *P* = 0.17, I^2^ = 33.6%) was discovered. However, in patients who were bTMB low, significant heterogeneity was observed (Q = 28.44, *P* < 0.01, I^2^ = 78.9%).

In the subgroup analysis of bTMB-high patients, across all subgroups (line of therapy, therapy regimen, type of NGS panel, PD-L1 expression, and cutoff value), patients who received PD-1/PD-L1 inhibitors had notably better PFS than those who received CT. The subgroup analysis of bTMB-low patients showed that patients receiving PD-1/PD-L1 inhibitor therapy had unimproved PFS compared with patients who received CT, regardless of line of therapy, type of NGS panel, PD-L1 expression and cutoff value. However, bTMB-low patients who received anti-PD-1 plus CT combination therapy showed significantly better PFS than CT patients (Table 3).

**Table 3:** Subgroup analyses of PFS in bTMB high and bTMB low NSCLC patients

Variable	No. 0f cohorts	No. of patients	bTMB high			bTMB low		
		bTMB high/bTMB low	Pooled HR (95% CI)	***P***	***I***^**2**^ (%)	Pooled HR (95% CI)	***P***	***I***^**2**^ (%)
**Line of therapy**								
1	5	528/1271	0.51 (0.37–0.70)	< 0.01	50.5	0.96 (0.66–1.39)	0.83	85.4
≥ 2	2	221/573	0.63 (0.47–0.84)	< 0.01	0	1.01 (0.84–1.20)	0.94	0
**Therapy regimen**								
Anti-PD-L1 vs CT	4	455/1269	0.65 (0.53–0.80)	< 0.01	0	1.05 (0.93–1.19)	0.4	0
Anti-PD-L1 plus anti-CTLA-4 vs CT	1	134/389	0.53 (0.34–0.82)	< 0.01	N/A	1.55 (1.23–1.95)	< 0.01	N/A
Anti-PD-1 plus CT vs CT	2	160/186	0.34 (0.22–0.51)	< 0.01	0	0.52 (0.36–0.74)	< 0.01	0
**Type of NGS panel**								
Foundation one	3	308/875	0.61 (0.47–0.78)	< 0.01	0	1.00 (0.87–1.16)	0.95	0
Gardant OMNI	3	379/920	0.53 (0.34–0.83)	< 0.01	67	0.99 (0.57–1.73)	0.99	92
OncoScreen Plus	1	62/49	0.30 (0.13–0.68)	< 0.01	N/A	0.63 (0.25–1.60)	0.33	N/A
**PD-L1 expression**								
Unselected for PD-L1	6	662/1542	0.54 (0.42–0.70)	< 0.01	44.6	0.99 (0.74–1.32)	0.93	82.2
PD-L1 positive	1	87/302	0.55 (0.33–0.92)	0.02	N/A	1.00 (0.78–1.29)	1.00	N/A
**Cut-off value (mut/meg)**								
16	3	308/875	0.61 (0.47–0.78)	< 0.01	0	1.00 (0.87–1.16)	0.95	0
Others	4	441/969	0.49 (0.32–0.74)	< 0.01	62.8	0.93 (0.56–1.54)	0.77	88.7

***mut/meg*** **mutation/mergbase,** ***N/A*** **not applicable,** ***95% CI*** **95% confidence interval,** ***HR*** **hazard ratio,** ***PFS*** **progression free survival**

### Relationship between bTMB level and objective response rate

**Fig. 4:**
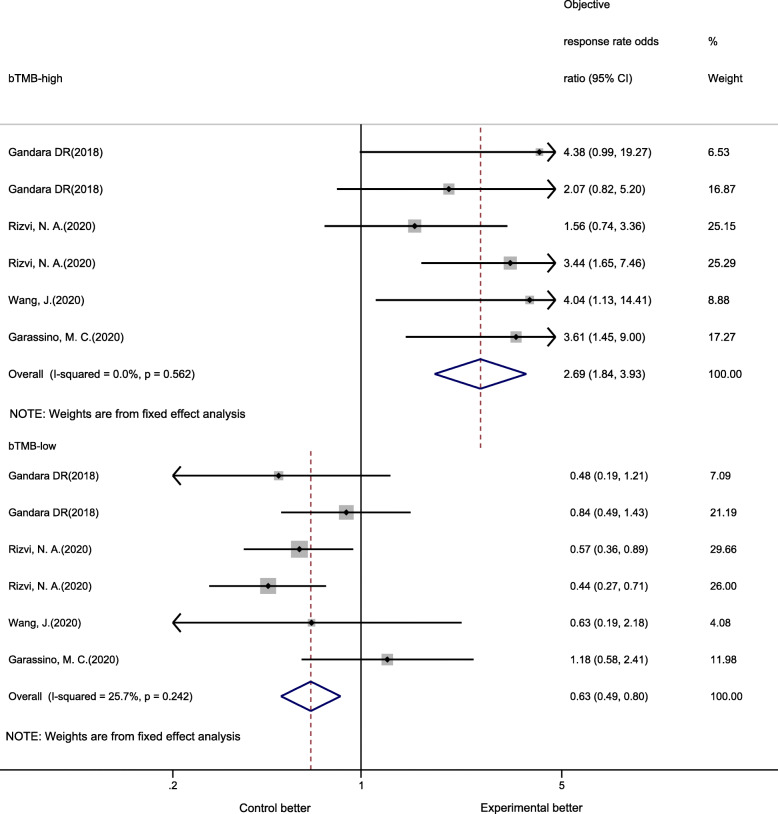
Forrest plot of odds ratios for objective response rate comparing PD-1/PD-L1 inhibitors with chemotherapy

Five trials (six cohorts) that included 2204 NSCLC patients evaluated the relationship between bTMB and the ORR of PD-1/PD-L1 inhibitor therapy. Compared with CT, PD-1/PD-L1 inhibitor therapy resulted in a notably better ORR in bTMB-high patients (ORR OR 2.69, 95% CI 1.84–3.93, *P* < 0.01), while bTMB-low patients showed a significantly worse ORR for PD-1/PD-L1 inhibitor therapy (ORR OR 0.63, 95% CI 0.49–0.80, *P* < 0.01) (Fig. 4). No substantial heterogeneity was found among individual studies among bTMB-high patients (Q = 3.92, *P* = 0.56, I^2^ = 0%), and bTMB-low patients (Q = 6.73, *P* = 0.24, I^2^ = 25.7%).

In subgroup analyses, across all subgroups (line of therapy, therapy regimen, type of NGS panel, PD-L1 expression and cutoff value), bTMB-high patients receiving PD-1/PD-L1 inhibitors had evidently better ORR than CT patients; bTMB-low patients receiving PD-1/PD-L1 inhibitors showed worse or unimproved ORR than CT patients (Table 4).

**Table 4:** Subgroup analyses of ORR in bTMB high and bTMB low NSCLC patients

Variable	No. 0f cohorts	No. of patients	bTMB high			bTMB low		
		bTMB high/bTMB low	Pooled HR (95% CI)	***P***	***I***^**2**^ (%)	Pooled HR (95% CI)	***P***	***I***^**2**^ (%)
**Line of therapy**								
1	4	441/969	2.55 (1.16–5.59)	< 0.01	0	0.62 (0.41–0.93)	0.02	41
≥ 2	2	221/573	2.74 (1.75–4.30)	0.02	5.9	0.73 (0.45–1.18)	0.19	4.9
**Therapy regimen**								
Anti-PD-L1 vs CT	3	368/967	1.98 (1.15–3.41)	0.02	0	0.64 (0.47–0.89)	< 0.01	0
Anti-PD-L1 plus anti-CTLA-4 vs CT	1	134/389	3.44 (1.62–7.31)	< 0.01	N/A	0.44 (0.27–0.71)	< 0.01	N/A
Anti-PD-1 plus CT vs CT	2	160/186	3.75 (1.79–7.88)	< 0.01	0	1.01 (0.54–1.86)	0.99	0
**Type of NGS panel**								
Foundation one	2	221/573	2.55 (1.16–5.59)	0.02	0	0.73 (0.45–1.18)	0.19	4.9
Gardant OMNI	3	379/920	2.62 (1.52–4.52)	< 0.01	28	0.63 (0.38–1.03)	0.06	60.6
OncoScreen Plus	1	62/49	4.04 (1.13–14.43)	0.03	N/A	0.63 (0.19–2.13)	0.46	N/A
**PD-L1 expression**								
Unselected for PD-L1	6	662/1542	2.69 (1.84–3.93)	< 0.01	0	0.64 (0.47–0.86)	< 0.01	25.7
**Cut-off value (mut/meg)**								
16	2	221/573	2.55 (1.16–5.59)	0.02	0	0.73 (0.45–1.18)	0.19	4.9
Others	4	441/969	2.74 (1.75–4.30)	< 0.01	0	0.64 (0.47–0.86)	0.02	41

***mut/meg*** **mutation/mergbase,** ***N/A*** **not applicable,** ***95% CI*** **95% confidence interval,** ***OR*** **odds ratio,** ***ORR*** **objective response rate**

### Meta-regression

Although grouped under the definition of NSCLC, squamous cell cancer and adenocarcinoma are different biological entities. Therefore, we performed a meta-regression to analyze the effect of histology on pooled HR and OR in bTMB-high and bTMB-low groups according to the proportion of patients with squamous cell histology in the cohorts: low (0–30%), intermediate (31–60%), and high (61–100%). Our results showed that the proportion of patients with squamous cell histology did not have a significant effect on pooled HR or OR in either the bTMB high or low groups (Supplementary Table 2).

### Risk of bias and sensitivity analysis

The results of the risk of bias assessment for eligible trials are provided in Supplementary Table 1. All trials conducted therapy allocation in a random way and were at low risk for selection bias and selective reporting; lack of blinding was the main issue affecting qualitybecause most trials were open-label. In addition, it should be noted that the BGB-A317–307 trial is still in progress, and only some of the results have been reported at present, which might lead to a risk of bias caused by incomplete data. Sensitivity analysis indicated that all results were stable (Supplementary Fig. S1–S3).

## Discussion

### Principal findings

The results of our meta-analysis showed that, compared with CT, PD-1/PD-L1 inhibitor therapy could decrease the risk of death by 38% in bTMB-high patients and by 14% in bTMB low patients. Moreover, bTMB-high patients achieved significantly better PFS and ORR from PD-1/PD-L1 inhibitor therapy than from CT, while bTMB-low patients showed unimproved PFS and worse ORR from PD-1/PD-L1 inhibitor therapy versus CT. The results were generally consistent across all subgroups. Interestingly, the subgroup analysis showed that, compared with CT, atezolizumab used as second-line therapy could significantly prolong the OS of bTMB-low patients; moreover, the combination therapy of PD-1 inhibitor and CT significantly improved the PFS of bTMB-low patients. However, it was difficult to draw any definite conclusion only from the data collected from the two trials. The sensitivity analysis indicated that all results were stable. There was significant heterogeneity in pooled OS and PFS in the bTMB-low group, which could be partially explained by the limited number of trials included and subgroup analyses by therapy regimen, type of NGS panel, line of therapy, PD-L1 selection status and different cutoff values. We calculated the pooled HR using a random effect model, which may help to minimize heterogeneity. In addition, to analyze the influence of tumor histology on the overall HR and OR, a meta-regression was conducted. The results indicated that the proportion of patients with squamous cell histology may not be a source of heterogeneity in either the bTMB high or low groups. However, the validity of the meta-regression might be reduced by the limited number of enrolled studies.

### Potential mechanisms

bTMB is defined as the number of mutations per megabase of ctDNA, which is released into plasma as a result of apoptosis and degradation of tumor cells. Recently, a bTMB detection assay was developed by Foundation Medicine, with the ability to count somatic mutations present at low allele frequency (0.5%) across 394 genes, starting from as little as 1% tumor content in at least 20 ng of ctDNA, and the accuracy comparison showed a good correlation between bTMB and tTMB values that were detected in the same samples [34]. Gandara et, al. demonstrated a positive correlation between TMB derived from pretreatment plasma from 259 patients, enrolled in the POPLAR and OAK trials, and matched tissue tested by FoundationOne CDx with a Spearman rank correlation of 0.64 (95% CI 0.56–0.71) [35]. The Guardant 360 and Guardant OMNI NGS panels were also designed for bTMB detection, and a positive correlation between tTMB (FoundationOne) and bTMB (Guardant OMNI) was found with a Pearson’s r of 0.7 [36]. It seems that bTMB may be able to serve as a feasible surrogate of tTMB in immunotherapy efficacy prediction.

However, with the deepening of research, many limitations of bTMB have been exposed, and bTMB by itself can be misleading if used directly as a predictive biomarker. The interesting part of bTMB is that it can be affected by several external factors and may vary greatly even between patients with the same tumor type [37]. Benign somatic heterogeneity caused by the accumulation of somatic mutations in nonneoplastic lesions during aging can be an important confounding factor [38]. Mutations propagating from adult stem cells to daughter cells during self-renewal may also have a significant effect on TMB detection [39]. Another challenge in ctDNA-based investigations is the lack of understanding of the release of ctDNA from cells into circulation. Previous studies have suggested that ctDNA is derived mainly from apoptotic tumor cells, while several recent studies have demonstrated a wider origin of ctDNA. For example, Razavi et al. applied a high-intensity sequencing assay to analyze plasma cfDNA and matched white blood cell (WBC) genomic DNA at a comparable raw depth, and their results revealed that a large proportion of mutations in the cfDNA of tumor patients may not originate from cancer cells but from malignant hemopoiesis. This finding indicated that most currently used clinical cfDNA-based assays that do not take the result of white blood cell (WBC) sequencing into account may cause substantial errors, and incorporating matched WBC sequencing into cfDNA-based clinical assays is necessary [40].

Additionally, the bTMB value can be different from tissues, which is why it is not truly ideal for clinical implementation, as high bTMB can be associated with low tTMB in half of the cases, although a correlation between blood and tissue-based determinations still exists [21, 41]. This finding may be partially explained by tumor heterogeneity, patient stage at the time of biopsy, tissue purity, time between tumor tissue and plasma sampling, etc. [29] Of course, a great deal of noise is contributed by genomic materials not shed from the tumor of interest, which is a common challenge in ctDNA-based investigations [42].

The Evaluation of Genomic Applications in Practice and Prevention (EGAPP) has proposed three prerequisite criteria for the adoption of a tumor biomarker test in clinical practice; these criteria are analytical validity (accuracy, reliability, and repeatability of the test), clinical validity (the ability to divide patients into different groups with quite different clinical outcomes), and clinical utility (if the clinical outcomes are improved for patients who received the test compared with those of patients who did not) [43]. At present, many of the blood-based assays used are polymerase chain reaction (PCR)-based or NGS-delivered. However, NGS panels have not been analytically or clinically validated, and little evidence of the clinical utility of ctDNA assays has been found [44]. In a recent study, researchers applied two different commercial ctDNA test assays (Guardant 360 and PlasmaSELECT) to assess the same batch of plasma samples. Their results showed that complete concordance between two assays was observed in only 9 of 34 samples tested [45]. This incongruence raised major concerns on the quality, reproducibility and therefore validity of these tests. It must be admitted that bTMB is largely an unvalidated biomarker, missing key points in biomarker development and appearing abruptly in clinical trials; not being implemented prior to prospective validation [46]. In addition, the situation is the same for tTMB: it was an exploratory marker. When utilized to enroll patients, tTMB failed to demonstrate predictive value [47]. Further sophisticated multicenter studies are desperately needed to conduct clinical and analytical validations and establish the relative regulatory guidelines to guide the future application of ctDNA-based biopsies.

At present, the several NGS panels that are commonly used all use different sequences, gene targets, and thresholds, while few studies have conducted cross-platform comparisons of NGS panels, which may result in a lack of harmonization between their results [48]. In addition, the computational algorithms for tTMB and bTMB are different, as the tTMB algorithm includes both insertions and deletions (indels) and single nucleotide variants (SNVs), while the bTMB calculation is based on SNVs only, indicating that this assay may miss some patients with high indels but low SNVs [29]. In addition, Gandara et al. found that in patients with high TMB (> 30 mutations/sample), in both bTMB and tTMB assays, a quarter of variants detected were unique to the tissue, and a third were unique to the blood, which also stressed the fundamental technical differences in sample characteristics and computational pipelines between bTMB and tTMB detection [29].

Moreover, it has been demonstrated that the maximum somatic allele frequency (MSAF) of ctDNA could provide additional predictive value for bTMB. MSAF, defined as the maximum allele frequency of the somatic mutations in ctDNA, could reflect the amount of ctDNA in blood. Generally, the detection of bTMB is based on the amount of ctDNA, which is a negative prognostic factor of PD-1/PD-L1 inhibitor therapy [42]. Researchers found that patients with a high bTMB, who are expected to achieve favorable clinical effects from PD-1/PD-L1 inhibitor therapy, are prone to having a high level of ctDNA, which is related to poor outcomes, resulting in a paradox [49]. Wang et al. [50] divided bTMB into two distinct parts according to MSAF: (1) mutation counts with a high allele frequency (HAF-bTMB) which would increase with MSAF (strongly correlated with the ctDNA amount); and (2) low allele frequency (LAF-bTMB), which does not change with the MSAF (does not correlate with the ctDNA amount). Then, they observed a trend toward worse OS, PFS, and ORR in HAF-bTMB-high patients than in LAF-bTMB-high patients, indicating that bTMB, adjusted for AF, can serve as a more reliable predictor for PD-1/PD-L1 inhibitor therapy.

### Comparison with previous studies and meta-analyses

To date, there is still no meta-analysis evaluating the association between PD-1/PD-L1 inhibitor therapy outcomes and bTMB levels in cancer patients. In addition, because it is generally recognized that low TMB levels are associated with weak efficacy of PD-1/PD-L1 inhibitors, only a few studies have assessed the efficacy of PD-1/PD-L1 inhibitors with CT in bTMB-low populations. A post hoc analysis based on the data from the POPLAR and OAK trials by Gandara et al. initially explored the role of bTMB in the immunotherapy response, and the results showed that higher bTMB was associated with improved OS, PFS and ORR among NSCLC patients receiving atezolizumab monotherapy; moreover, bTMB-low patients could also obtain a long-term survival benefit from PD-1/PD-L1 inhibitor therapy [29]. In the retrospective analysis from the MYSTIC trial, the same conclusion was drawn in patients treated with durvalumab with or without tremelimumab compared with treatment with CT; however, no survival benefit in bTMB-low patients treated with combination therapy was found [31]. B-F1RST was the first prospective multicenter study to assess the predictive role of bTMB for atezolizumab response in NSCLC patients. Patients in the high-bTMB group had numerical benefits in PFS and OS compared with those in the bTMB low group [24]. KEYNOTE-189 demonstrated that bTMB was also associated with the outcome of the combination of pembrolizumab and CT [33].

Although these studies obtained results that are generally consistent, the bTMB cutoff values in these studies are different (POPLAR, OAK, B-F1RST = 16 mut/meg; MYSTIC = 20 mut/meg; KEYNOTE-189 = 15 mut/meg), and the best cutoff value for the definition of bTMB-high and bTMB-low patients still requires further studies to reach a convincing conclusion. Interestingly, Nie et al. [51] revealed a new strategy by dividing patients into bTMB-high, bTMB-medium, and bTMB-low groups, and found that patients with high and low bTMB had longer OS and PFS than patients with medium bTMB, which suggested a nonlinear association between bTMB and survival outcomes in patients treated with PD-1/PD-L1 inhibitors. However, their study only had a moderate sample size; inaddition, the proportion of patients with KEAP1 and STK11 mutations in the bTMB-low group was higher than that in the bTMB-medium group, and bTMB-low patients may receive more benefit from PD-1/PD-L1 inhibitor therapy due to the higher frequency of STK11 and KEAP1 mutations and a lower tumor burden, thus overestimating the therapeutic efficacy [52]. Their results should be further confirmed by studies with larger sample sizes and more sophisticated designs, but the three-tier classification scheme may improve the accuracy of bTMB detection.

However, it must be clarified that RCTs have thus far failed to show a survival benefit when stratifying patients by TMB, and only post hoc analyses have shown some predictive value [17, 53, 54]. In fact, a meta-analysis of randomized trials has shown a lower value of TMB vs. other clinically implemented biomarkers for the prediction of a benefit from anti-PD-1/PD-L1 treatment, e.g., PD-L1 immunohistochemistry (IHC) [55]. It has been demonstrated that compared with PD-L1 IHC, TMB, autoantibody, or gene expression profile (GEP) alone, combining two or more biomarkers could achieve a better sensitivity and specificity for identifying patients most likely to respond to anti-PD-1/PD-L1 therapy [56]. Integration of TMB with a host of other biomarkers may result in more effective prediction of response to ICI therapy in the future.

#### Limitations and future study directions

In this study, we adopted OS, PFS, and ORR as our endpoints and investigated the efficacy of bTMB as a predictor of both long-term and short-term benefits of PD-1/PD-L1 inhibitor therapy. However, this study is restricted by many limitations, and the results are explorative and should be interpreted with caution.

First, only six trials were enrolled in this meta-analysis, which is quite limited, and a funnel plot cannot detect publication bias effectively when the number of studies included is less than ten [28]. In addition, for some subgroup analyses in this study, only 2 trials were included, which might increase the probability of selection bias. Although most data pooled in this study were from phase 3 RCTs, it should be noted that all these data are from analyses that were not preplanned, exploratory, conducted on smaller subsets, not part of the enrollment criteria nor of the secondary endpoints, or only conducted on one of the multiple explorative endpoints. This means that the quality of the available data is the same as that obtained from retrospective analyses, although they were obtained from prospective cohorts. Further RCTs with tTMB or bTMB status as a component of the enrollment criteria are required. Second, due to the use of different cutoff points and gene panel platforms for bTMB detection, the reproducibility of the threshold was significantly affected. The influence of various external factors may also reduce the utility of bTMB detection. In the present analysis, only a subset of patients enrolled in the POPLAR and OAK (32.6%), MYSTIC (43.5%), and KEYNOTE-189 (60.4%) studies were able to be included in the pairwise comparison of bTMB and tTMB, although a positive correlation between bTMB and tTMB was demonstrated. Further prospective RCTs using tTMB and bTMB to investigate the predictive validity for ICI therapy response and concordance across testing platforms are needed. Development of a bTMB algorithm is also required, the MSAF of ctDNA should be considered, and the indels of ctDNA should also be taken into account to enhance the consistency between bTMB and tTMB assays. In addition, the threshold, as one of the most critical issues in bTMB detection, still requires further studies to become standardized. Another limitation of the bTMB assay is that a minimum amount of ctDNA with sufficient quality in the blood for optimal assay performance is required, and this might not always be accessible, indicating that more sensitive NGS panels are still needed [57].

Third, a few important clinical characteristics, which have been proven to be responsible for the efficacy of PD-1/PD-L1 inhibitor therapy, such as sex, age, combination therapy, and residential area of patients could not be analyzed in this study because the data were not available. Moreover, the cohorts from the OAK and POPLAR studies included in this analysis were mixed oncogene addicted (EGFR, KRAS, and ALK) and nonaddicted, which may affect the response to ICI therapy and result in an overestimation of bTMB [58, 59].

Fourth, toxicity is always an important factor in choosing therapy options; however, we were not able to analyze this issue here due to the lack of studies focused on the relationship between bTMB levels and the toxicity of PD-1/PD-L1 inhibitor therapy. More trials are needed to evaluate whether bTMB could be used in the assessment of immune-related adverse events during ICI treatment.

In addition, several analyses have favored the hypothesis that PD-L1 and TMB are independent biomarkers of both mono- and combined ICI therapies. These studies suggested that TMB may complement PD-L1 expression assessment and help to identify a subgroup of PD-L1-low or PD-L1-nonexpressing patients who may benefit from single-agent or combined ICI therapy [60, 61]. However, the present data are insufficient to support a systematic evaluation of the predictive efficacy of combined detection of PD-L1 and bTMB; thus, further RCTs evaluating the efficacy of a combination of multiple biomarkers should be considered.

In summary, although compared with conventional tissue biopsy calculating TMB from blood is more accessible, noninvasive, and contemporaneous and may be less vulnerable to potential sampling biases caused by single-site tissue biopsies, validated assays have yet to be developed, and tissue biopsy is still the standard method for TMB detection.

## Conclusion

The results of our meta-analysis suggest that the efficacy of PD-1/PD-L1 inhibitors for NSCLC therapy might be bTMB level dependent. Compared with CT, PD-1/PD-L1 inhibitors resulted in significantly improved OS, PFS and ORR among bTMB-high patients, while bTMB-low patients showed unimproved OS and PFS and worse ORR from PD-1/PD-L1 inhibitor therapy.

## Supplementary Information

**Additional file 1.**

## Data Availability

The data that support the findings of this study are openly available.

## Abbreviations

PD-1: programmed cell death 1
PD-L1: programmed cell death ligand 1
NSCLC: non-small cell lung cancer
bTMB: blood-based tumor mutation burden
HR: hazard ratio
OS: overall survival
PFS: progression free survival
OR: odds ratio; ORR objective response rate
CT: chemotherapy
TMB: tumor mutation burden
tTMB: tissue-based tumor mutation burden
ctDNA: circulating tumor deoxyribonucleic acid
PRISMA: Preferred Reporting Items for Systematic Reviews and Meta-analyses
RCTs: randomized controlled trials
95% CI: 95% confidence intervals
MSAF: maximum somatic allele frequency
HAF: high allele frequency
LAF: low allele frequency
SNVs: single nucleotide variants
indels: insertions and deletions
Exp: experimental group
BEP: bTMB-evaluable population
Con: control group
mut/meg: mutation/mergbase
NR: not reported
