# Supplementary material for: The relationship between blood-based tumor mutation burden level and efficacy of PD-1/PD-L1 inhibitors in advanced non-small cell lung cancer: a systematic review and meta-analysis
Source: BMC Cancer. 2021 Nov 13;21:1220. doi: 10.1186/s12885-021-08924-z (PMC8590772; doi:10.1186/s12885-021-08924-z)
Supplement: Supplementary file 1 — Additional file 1. [file 12885_2021_8924_MOESM1_ESM.docx]

**Supplementary Materials**

**Search strategy**

**PubMed: 83 results**

(((("lung cancer*"[Title/Abstract] OR "lung carcinoma*"[Title/Abstract] OR "lung neoplasm*"[Title/Abstract] OR "lung tumor*"[Title/Abstract] OR "lung tumour*"[Title/Abstract]) AND ("non-small cell*"[Title/Abstract] OR "nonsmall cell*"[Title/Abstract])) OR (("Carcinoma, Non-Small-Cell Lung"[Mesh]) OR (NSCLC[Title/Abstract]))) AND ((("Liquid Biopsy"[Mesh] OR "Cell-Free Nucleic Acids"[Mesh] OR "Circulating Tumor DNA"[Mesh]) OR ("cell free"[Title/Abstract] OR circulat*[Title/Abstract] OR extracellular*[Title/Abstract] OR blood*[Title/Abstract] OR plasma*[Title/Abstract] OR serum*[Title/Abstract] OR "liquid biops*"[Title/Abstract] OR cirDNA[Title/Abstract] OR cfDNA[Title/Abstract] OR ctDNA[Title/Abstract])) AND (TMB[Title/Abstract] OR TML[Title/Abstract] OR "mutation burden"[Title/Abstract] OR "mutational burden"[Title/Abstract] OR "mutation load"[Title/Abstract] OR "mutational load"[Title/Abstract]))) AND (("durvalumab" [Supplementary Concept] OR "avelumab" [Supplementary Concept] OR "atezolizumab" [Supplementary Concept] OR "pembrolizumab" [Supplementary Concept] OR "Nivolumab"[Mesh] OR "Programmed Cell Death 1 Receptor"[Mesh] OR "Programmed Cell Death 1 Ligand 2 Protein"[Mesh] OR "CD274 protein, human" [Supplementary Concept] OR "B7-H1 Antigen"[Mesh] OR "Immunotherapy"[Mesh] OR "Immune Checkpoint Inhibitors"[Mesh]) OR (Durvalumab[Title/Abstract] OR MEDI4736[Title/Abstract] OR MEDI-4736[Title/Abstract] OR Imfinzi[Title/Abstract] OR avelumab[Title/Abstract] OR MSB0010682[Title/Abstract] OR bavencio[Title/Abstract] OR MSB0010718C[Title/Abstract] OR atezolizumab[Title/Abstract] OR MPDL3280A[Title/Abstract] OR Tecentriq[Title/Abstract] OR RG7446[Title/Abstract] OR pembrolizumab[Title/Abstract] OR SCH-900475[Title/Abstract] OR Keytruda[Title/Abstract] OR MK3475[Title/Abstract] OR MK 3475[Title/Abstract] OR lambrolizumab[Title/Abstract] OR Nivolumab[Title/Abstract] OR Opdivo[Title/Abstract] OR ONO-4538[Title/Abstract] OR MDX-1106[Title/Abstract] OR BMS-936558[Title/Abstract] OR ticilimumab[Title/Abstract] OR PD1[Title/Abstract] OR PD-1[Title/Abstract] OR "Programmed Cell Death 1"[Title/Abstract] OR CD279[Title/Abstract] OR "Programmed Cell Death 1 Ligand 2"[Title/Abstract] OR CD273[Title/Abstract] OR PDL2[Title/Abstract] OR PD-L2[Title/Abstract] OR B7DC[Title/Abstract] OR B7-DC[Title/Abstract] OR PDL1[Title/Abstract] OR "programmed cell death 1 ligand 1"[Title/Abstract] OR PD-L1[Title/Abstract] OR B7-H1[Title/Abstract] OR B7H1[Title/Abstract] OR PDCD1LG1[Title/Abstract] OR CD274[Title/Abstract] OR immunother*[Title/Abstract] OR immunization[Title/Abstract] OR immunisation[Title/Abstract] OR "Immune Checkpoint Inhibitor*"[Title/Abstract] OR ICI[Title/Abstract] OR ICIs[Title/Abstract] OR ICB[Title/Abstract] OR ICBs[Title/Abstract] OR "immune checkpoint block*"[Title/Abstract]))

**Embase: 393 results**

#1： 'durvalumab'/exp

#2： 'avelumab'/exp

#3： 'atezolizumab'/exp

#4： 'pembrolizumab'/exp

#5： 'nivolumab'/exp

#6： 'ticilimumab'/exp

#7： 'programmed death 1 receptor'/exp

#8： 'programmed death 1 ligand 2'/exp

#9：'programmed death 1 ligand 1'/exp

#10：'immunotherapy'/exp

#11： durvalumab:ab,ti OR medi4736:ab,ti OR imfinzi:ab,ti

#12： avelumab:ab,ti OR bavencio:ab,ti OR msb0010718c:ab,ti

#13： atezolizumab:ab,ti OR mpdl3280a:ab,ti OR tecentriq:ab,ti OR rg7446:ab,ti

#14： pembrolizumab:ab,ti OR 'sch900475':ab,ti OR keytruda:ab,ti OR mk3475:ab,ti OR lambrolizumab:ab,ti

#15： nivolumab:ab,ti OR opdivo:ab,ti OR 'ono4538':ab,ti OR 'mdx1106':ab,ti OR 'bms936558':ab,ti

#16： pd1:ab,ti OR 'pd-1':ab,ti OR 'programmed cell death 1':ab,ti OR cd279:ab,ti

#17： 'programmed cell death 1 ligand 2':ab,ti OR cd273:ab,ti OR pdl2:ab,ti OR 'pd-l2':ab,ti OR b7dc:ab,ti OR 'b7 dc':ab,ti

#18： pdl1:ab,ti OR 'programmed cell death 1 ligand 1':ab,ti OR 'pd-l1':ab,ti OR 'b7 h1':ab,ti OR b7h1:ab,ti OR pdcd1lg1:ab,ti OR cd274:ab,ti

#19： immunother*:ab,ti OR immuni?ation:ab,ti OR 'immune checkpoint inhibitor*':ab,ti OR ici:ab,ti OR icis:ab,ti OR 'immune checkpoint block*':ab,ti OR icbs:ab,ti OR icb:ab,ti

#20： #1 OR #2 OR #3 OR #4 OR #5 OR #6 OR #7 OR #8 OR #9 OR #10 OR #11 OR #12 OR #13 OR #14 OR #15 OR #16 OR #17 OR #18 OR #19

#21： 'cell free nucleic acid'/exp

#22： 'liquid biopsy'/exp

#23： 'circulating tumor dna'/exp

#24： 'cell free':ab,ti OR circulat*:ab,ti OR extracellular*:ab,ti OR blood*:ab,ti OR plasma*:ab,ti OR serum*:ab,ti

#25： cirdna:ab,ti OR cfdna:ab,ti OR ctdna:ab,ti

#26： #21 OR #22 OR #23 OR #24 OR #25

#27: 'mutation burden':ab,ti OR 'mutational burden':ab,ti OR 'mutation load':ab,ti OR 'mutational load':ab,ti OR tmb:ab,ti OR tml:ab,ti

#28： #26 AND #27

#29: 'lung cancer'/exp

#30: 'non small cell lung cancer'/exp

#31: 'lung cancer*':ab,ti OR 'lung carcinoma*':ab,ti OR 'lung neoplasm*':ab,ti OR 'lung tumor*':ab,ti OR 'lung tumour*':ab,ti

#32: 'non small cell*':ab,ti OR 'nonsmall cell*':ab,ti OR nsclc:ab,ti

#33: #29 OR #30

#34: #31 AND #32

#35: #33 OR #34

#36: #35 AND #28 AND #20

**Cochrane : 122 results**

#1： MeSH descriptor: [Nivolumab] explode all trees

#2： MeSH descriptor: [Programmed Cell Death 1 Receptor] explode all trees

#3： MeSH descriptor: [Programmed Cell Death 1 Ligand 2 Protein] explode all trees

#4： MeSH descriptor: [B7-H1 Antigen] explode all trees

#5： MeSH descriptor: [Immunotherapy] explode all trees

#6： (Durvalumab OR MEDI4736 OR MED-I4736 OR Imfinzi):ti,ab,kw

#7： (avelumab OR MSB0010682 OR bavencio OR MSB0010718C OR MSB-0010682 OR MSB-0010718C):ti,ab,kw

#8： (atezolizumab OR MPDL3280A OR Tecentriq OR RG7446 OR MPDL-3280A OR RG-7446):ti,ab,kw

#9： (pembrolizumab OR SCH900475 OR lambrolizumab OR Keytruda OR MK3475 OR MK-3475 OR SCH-900475):ti,ab,kw

#10： (Nivolumab OR Opdivo OR ONO4538 OR BMS936558 OR MDX1106 OR MDX-1106 OR ONO-4538 OR BMS-936558):ti,ab,kw

#11： (PD1 OR PD-1 OR "Programmed Cell Death 1" OR CD279):ti,ab,kw

#12： ("Programmed Cell Death 1 Ligand 2"):ti,ab,kw

#13： (CD273 OR PDL2 OR PD-L2 OR B7DC):ti,ab,kw

#14： (“programmed cell death 1 ligand 1”):ti,ab,kw

#15： (PDL1 OR PD-L1 OR B7H1 OR PDCD1LG1 OR CD274):ti,ab,kw

#16： (immunother* OR immunization OR immunisation):ti,ab,kw

#17： ((Immune Checkpoint) NEXT/2 (Inhibitor* OR block*)):ti,ab,kw

#18： (ICI OR ICIs OR ICB OR ICBs):ti,ab,kw

#19： #1 OR #2 OR #3 OR #4 OR #5 OR #6 OR #7 OR #8 OR #9 OR #10 OR #11 OR #12 OR #13 OR #14 OR #15 OR #16 OR #17 OR #18

#20： MeSH descriptor: [Cell-Free Nucleic Acids] explode all trees

#21： MeSH descriptor: [Liquid Biopsy] explode all trees

#22： MeSH descriptor: [Circulating Tumor DNA] explode all trees

#23： (“cell free” OR circulat* OR extracellular* OR blood* OR plasma* OR serum*):ti,ab,kw

#24： (cirDNA OR cfDNA OR ctDNA):ti,ab,kwx

#25： #20 OR #21 OR #22 OR #23 OR #24

#26： (“mutation burden” OR “mutation load” OR “mutational burden” OR “mutational load” OR TMB OR TML):ti,ab,kw

#27： #31 AND #32

#28： #33 AND #25

#29: MeSH descriptor: [Lung Neoplasms] explode all trees

#30: MeSH descriptor: [Carcinoma, Non-Small-Cell Lung] explode all trees

#31: ("nonsmall cell*" OR "non small cell*" OR NSCLC):ti,ab,kw

#32: (“lung cancer*” OR “lung carcinoma*” OR “lung neoplasm*” OR “lung tumor*” OR “lung tumour*”):ti,ab,kw

#33: #31 AND #32

#34: #29 OR #30 OR #33

**Web of Science: 50 results**

#1: AB=(Durvalumab OR MEDI4736 OR MEDI-4736 OR Imfinzi OR avelumab OR MSB0010682 OR bavencio OR MSB0010718C OR atezolizumab OR MPDL3280A OR Tecentriq OR RG7446 OR pembrolizumab OR SCH-900475 OR Keytruda OR MK3475 OR MK 3475 OR lambrolizumab OR Nivolumab OR Opdivo OR ONO-4538 OR MDX-1106 OR BMS-936558 OR PD1 OR PD-1 OR "Programmed Cell Death 1" OR CD279 OR "Programmed Cell Death 1 Ligand 2” OR CD273 OR PDL2 OR PD-L2 OR B7DC OR B7-DC OR PDL1 OR “programmed cell death 1 ligand 1” OR PD-L1 OR B7-H1 OR B7H1 OR PDCD1LG1 OR CD274 OR immunother* OR immunization OR immunisation OR "Immune Checkpoint Inhibitor*” OR ICI OR ICIs OR ICB OR ICBs OR “immune checkpoint blocker*”)

#2: AB=(“cell free” OR circulat* OR extracellular* OR blood* OR plasma* OR serum* OR “liquid biops*” OR cirDNA OR cfDNA OR ctDNA)

#3: AB=(TMB OR TML OR “mutation burden” OR “mutational burden” OR “mutation load” OR “mutational load”)

#4: #2 AND #3

#5: AB=(NSCLC OR “non small cell*” OR “nonsmall cell*”) AND AB=("lung cancer*" OR "lung carcinoma*" OR "lung neoplasm*" OR "lung tumor*" OR "lung tumour*")

#6: #1 AND #4 AND #5

Table S1. Quality assessment: risk of bias assessed by Cochrane Collaboration’s tool

| **Study ID** | **Year of publication** | **Sequence generation** | **Allocation concealment** | **Blinding** | **Incomplete outcome data** | **Selective reporting** | **Other source of bias** |
| --- | --- | --- | --- | --- | --- | --- | --- |
| POPLAR (NCT01903993) | 2018 | Low risk | Low risk | High risk | Low risk | Low risk | Low risk |
| OAK (NCT02008227) | 2018 | Low risk | Low risk | High risk | Low risk | Low risk | Low risk |
| MYSTIC (NCT02453282) | 2020 | Low risk | Low risk | High risk | Low risk | Low risk | Low risk |
| IMpower110 (NCT02409342) | 2020 | Low risk | Low risk | High risk | Low risk | Low risk | Low risk |
| BGB-A317-307 (NCT03594747) | 2020 | Low risk | Low risk | High risk | High risk | Low risk | Low risk |
| KEYNOTE-189 (NCT02578680) | 2020 | Low risk | Low risk | Low risk | Low risk | Low risk | Data from the abstract |

**Table S2. Meta-regression analysis of the influence of histology on clinical outcomes**

| **Clinical outcomes** | | **Coefficient** | **SE** | **-95% CI** | **+95% CI** | **p-value** |
| --- | --- | --- | --- | --- | --- | --- |
| bTMB high | OS | -0.0004 | 0.0078 | -0.022 | 0.021 | 0.96 |
|  | ORR | 0.0005 | 0.0082 | -0.022 | 0.023 | 0.96 |
|  | PFS | -0.0005 | 0.0059 | -0.016 | 0.015 | 0.93 |
| bTMB low | OS | 0.0079 | 0.0108 | -0.022 | 0.038 | 0.51 |
|  | ORR | -0.0063 | 0.0077 | -0.028 | 0.015 | 0.46 |
|  | PFS | 0.00162 | 0.0073 | -0.017 | 0.020 | 0.83 |


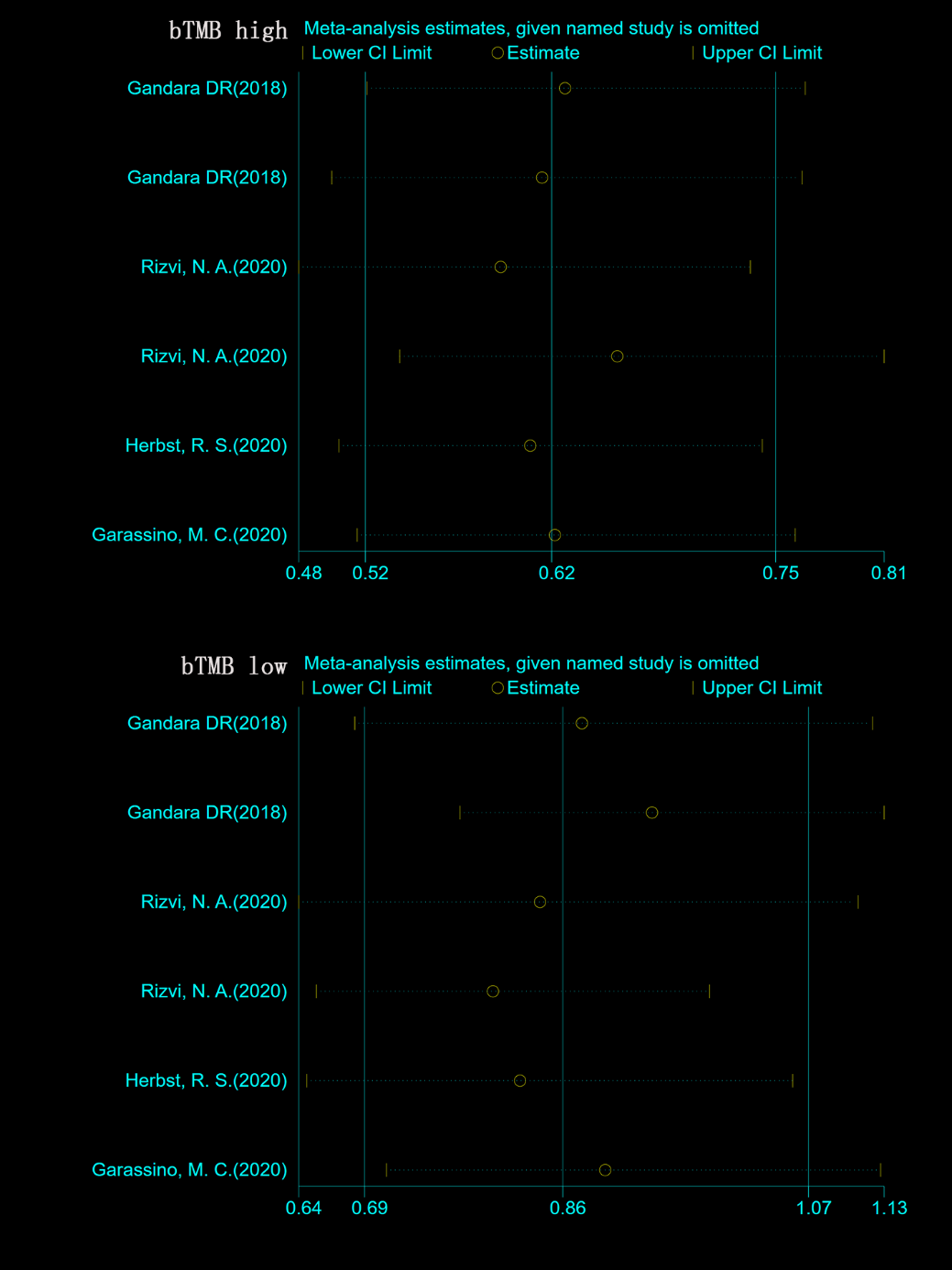


Figure S1: Sensitivity analyses of overall survival.


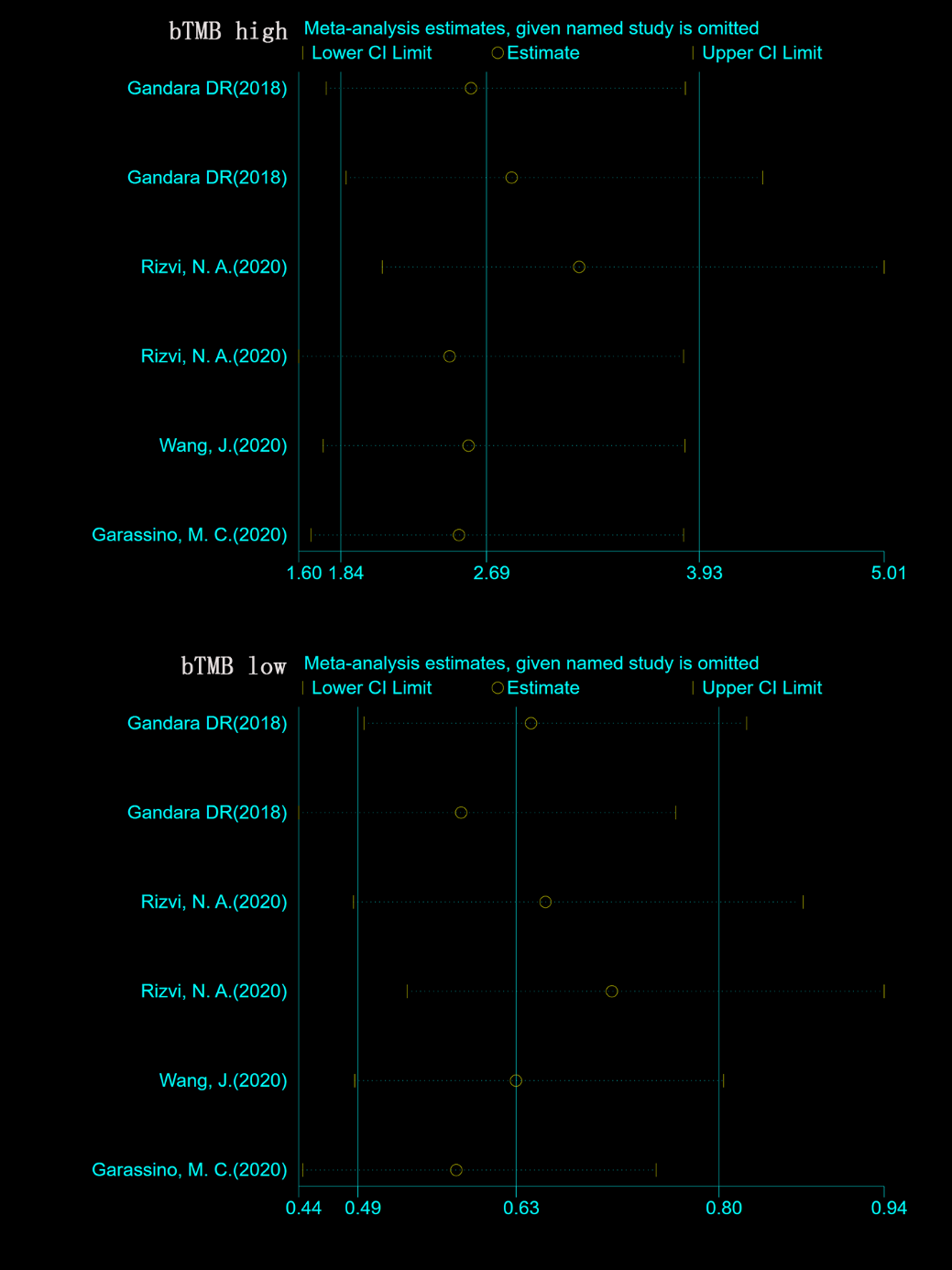


Figure S2: Sensitivity analyses of progression free survival.


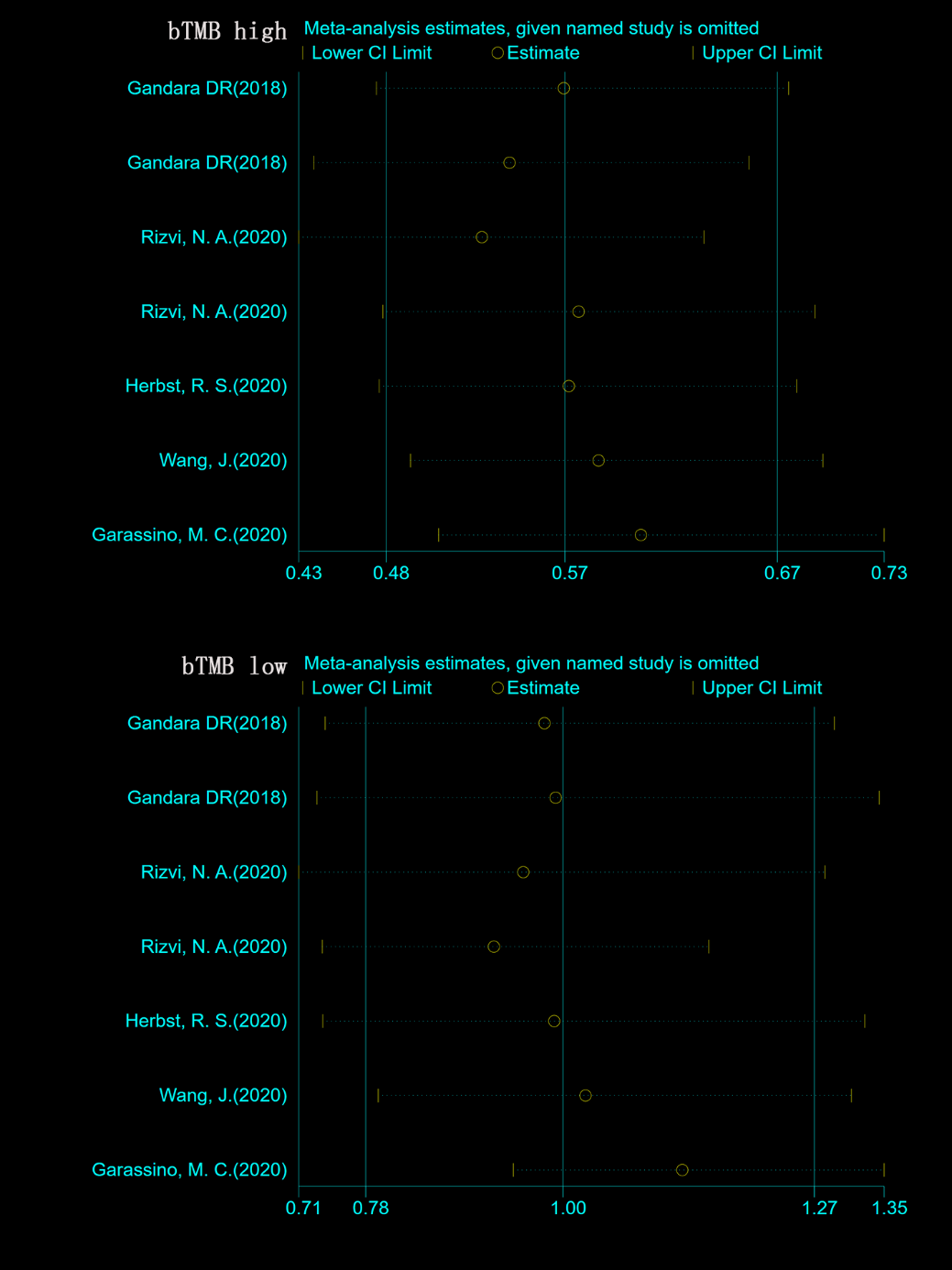


Figure S3: Sensitivity analyses of objective response rate.
